# Giant aneurysm of left circumflex artery branch with fistula to the coronary sinus: a case report

**DOI:** 10.1186/s13019-022-01950-3

**Published:** 2022-08-21

**Authors:** Yasuyuki Toyoda, Takahiro Takemura, Kazuaki Shiratori, Yoshikazu Yazaki, Takahiro Tachibana, Hirokazu Niitsu, Takashi Kunihara

**Affiliations:** 1 Department of Cardiovascular Surgery, Saku Central Hospital Advanced Care Center, 3400-28 Nakagomi Saku, Nagano, 385-0051 Japan; 2 Department of Cardiology, Saku Central Hospital Advanced Care Center, Nagano, Japan; 3 Department of Cardiac Surgery, Jikei University School of Medicine, Tokyo, Japan

**Keywords:** Coronary artery fistula, Coronary artery aneurysm, Aneurysmectomy

## Abstract

**Background:**

Aneurysm of a coronary artery branch with a fistula is extremely rare. Here, we present a case of giant aneurysm of the left circumflex artery branch with a fistula to the coronary sinus treated successfully with aneurysmectomy.

**Case presentation:**

A 58-year-old woman was referred to our hospital due to an abnormal pericardial mass found by multidetector computed tomography. Imaging examination revealed a dilated left circumflex artery branch with a 30-mm aneurysm. Coronary angiography confirmed a left circumflex artery branch aneurysm with a fistula to the coronary sinus. As percutaneous occlusion of the aneurysm by catheterization was considered unsuccessful, the aneurysm was resected, and the fistula was occluded surgically with excellent outcome. Pathological examination suggested that congenital factors may have contributed to the development of the aneurysm. Computed tomography showed no recurrence of the aneurysm at 1-year postoperative follow-up.

**Conclusions:**

We presented a case of giant aneurysm of the left circumflex artery branch with a fistula to the coronary sinus. This is the first report of the combination of a giant coronary artery branch aneurysm with a fistula to the coronary sinus. Surgical aneurysmectomy should be considered in such cases to avoid fatal aneurysmal complications.

## Background

Coronary artery fistula is an uncommon congenital malformation, with 10% of cases accompanied by a coronary artery aneurysm [1]. Aneurysm of a coronary artery branch with a fistula is extremely rare. Here, we present a case of giant aneurysm of the left circumflex artery (LCX) branch with a fistula to the coronary sinus (CS) and describe our successful surgical strategy.

## Case presentation

A 58-year-old asymptomatic woman was referred to our hospital due to an abnormal pericardial mass found incidentally on chest computed tomography (CT) during a medical check-up. On physical examination, she had normal heart sounds, no cardiac murmur, and no lung rales. She had a history of diabetes mellitus, hypertension, and hyperlipidemia, for which she was receiving appropriate medical therapy. She had no history of congenital heart disease, Kawasaki disease, or cardiac trauma, and no relevant family history.

## Investigation

**Fig. 1:**
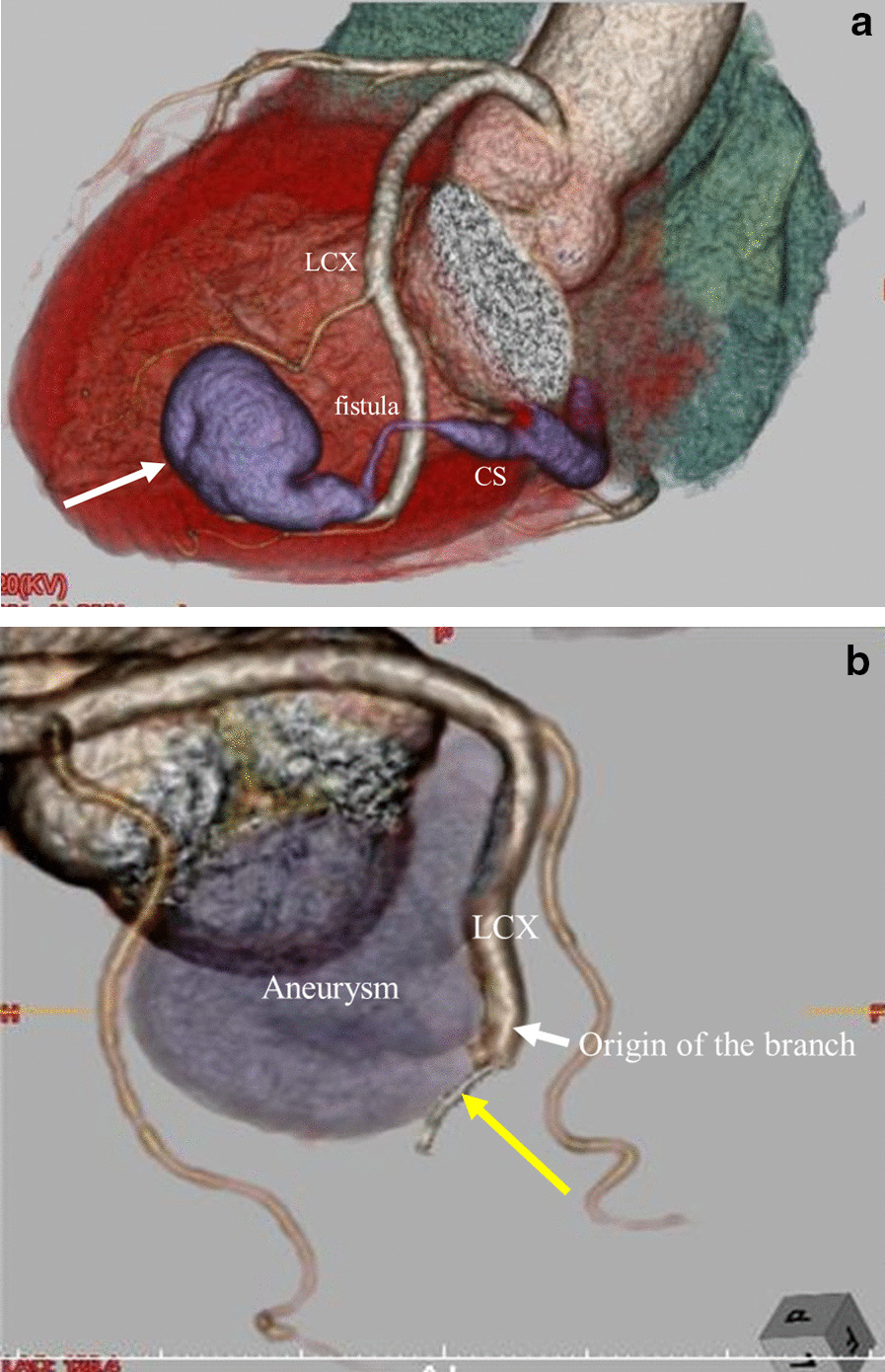
**a** Preoperative coronary artery computed tomography revealed a giant coronary branch aneurysm originating from near the terminal portion of the main LCX trunk with a fistula draining into the CS (white arrow). **b** The end of the main LCX was very thin (yellow arrow). CS, coronary sinus; LCX, left circumflex artery

**Fig. 2:**
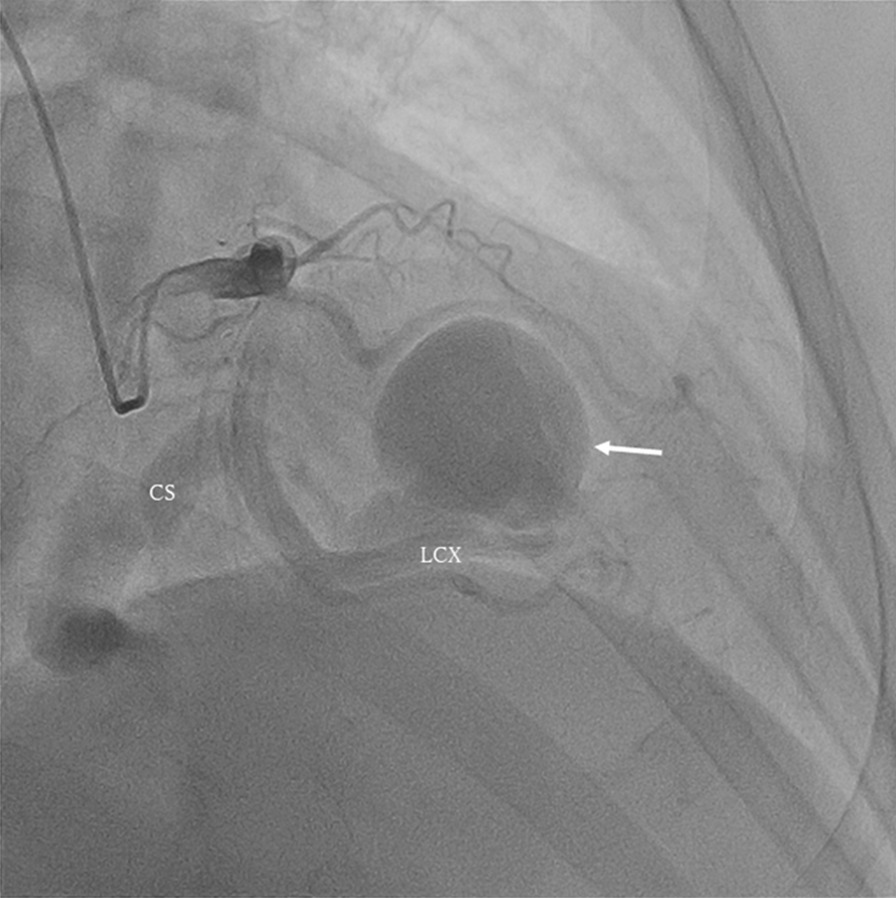
Preoperative coronary artery angiography showed that the abnormal aneurysm branched from the end of the LCX (white arrow). The aneurysm drained into the CS. CS, coronary sinus; LCX, left circumflex artery

Electrocardiography showed normal sinus rhythm and no significant ST-T changes. Chest X-ray showed neither cardiomegaly nor cardiac congestion. Transthoracic echocardiography demonstrated normal wall motion with left ventricular ejection fraction (LVEF) of 64%. Multidetector CT revealed a huge coronary branch aneurysm originating from close to the terminal portion of the main LCX trunk with a fistula directly draining to the CS (Fig. 1a, b). The end of the main LCX was very thin, indicating abundant blood flow to the coronary branch aneurysm. Coronary angiography showed a giant branch aneurysm arising from the LCX, whereas the left anterior descending artery and right coronary artery appeared normal (Fig. 2). The distal side of the aneurysm drained into the CS without a capillary bed, indicating the presence of a coronary artery fistula between the aneurysm and the CS. The trunk of the LCX itself was of normal caliber, but it became aneurysmal about 5 mm distal to its branch, measuring 30 mm in diameter. There were no side branches arising from the aneurysm. Percutaneous occlusion of the fistula by catheterization was initially considered to reduce the blood flow into the giant aneurysm, but this was difficult because cannulation was impossible. The heart team referred the patient for surgical closure.

## Management

**Fig. 3:**
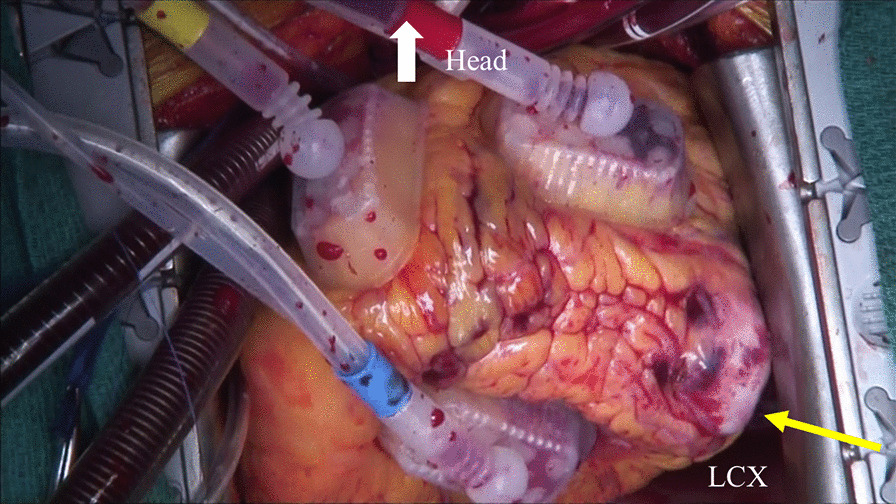
Operative view of the LCX branch aneurysm with fistula (yellow arrowhead). LCX, left circumflex artery

Surgical treatment was performed through a median sternotomy. Cardiopulmonary bypass was established using aortic cannulation and bicaval cannulation, and the aneurysm was found at the branch of the LCX (Fig. 3). After clamping of the ascending aorta, cardiac arrest was achieved by antegrade cardioplegia. The apex was lifted, the aneurysm was incised, and its proximal and distal orifices were closed with 5–0 polypropylene sutures. The aneurysm was excluded, and the patient was smoothly weaned from cardiopulmonary bypass. The operation time, cardiopulmonary bypass time, and aortic clamp time were 174, 71, and 25 min, respectively.

**Fig. 4:**
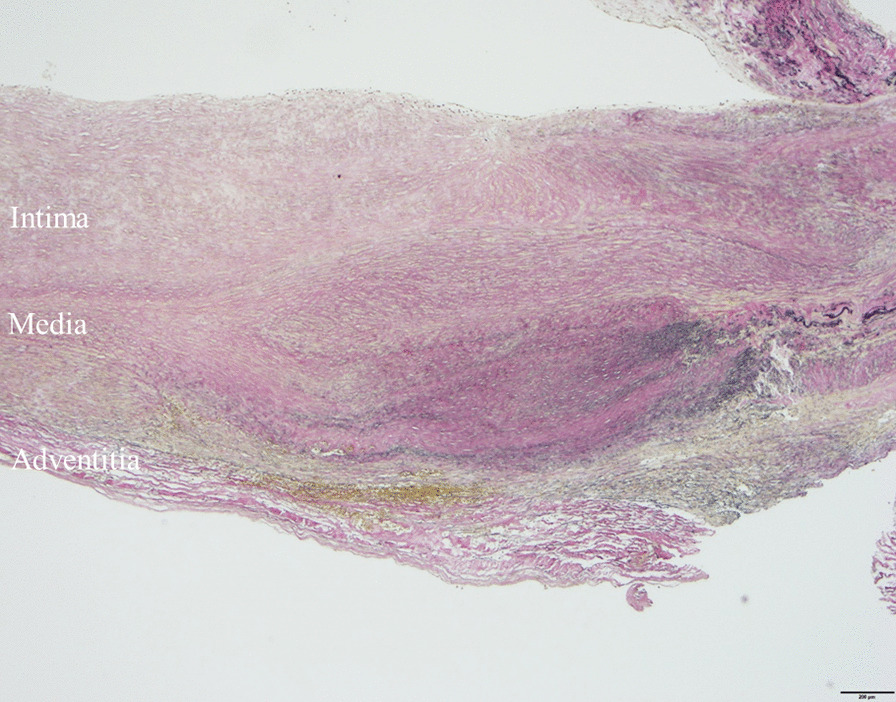
The resected left circumflex artery branch aneurysm specimen had a thickened intima with no hyaline arteriolosclerosis, thin fibrous media with small amounts of muscular components, and no internal elastic membrane. Stain: Elastin van Gieson

The postoperative course was uneventful with no clinically significant changes on electrocardiography. Postoperative blood examination revealed no relevant elevation of serum creatinine kinase MB level (max. 11 IU /L). Postoperative multidetector CT demonstrated absence of the fistula and no recurrence of the aneurysm. CT also showed no expansion of the LCX branch aneurysm at 1-year postoperative follow-up. Pathological examination of the surgical specimen revealed a thickened intima without hyaline arteriolosclerosis, thin media with minimal smooth muscle, and lack of an internal elastic membrane, which was markedly different from the typical media. These findings suggested that inherent weakness of the coronary branch artery wall may have contributed to the aneurysm (Fig. 4).

## Discussion and conclusions

Coronary artery aneurysm with an associated fistula (CAAAF) is a rare congenital malformation. Neufield et al. [2] first reported a surgical case of CAAAF in a 10-year-old girl in 1961. In 1963, Habermann et al. [3] reported a CAAAF in an adult patient who died of aneurysm rupture. The number of cases of CAAAF has gradually increased with the widespread adoption of coronary angiography but reports of this disease are still limited. Coronary artery branch aneurysm with a fistula is extremely rare. Hirose et al. [1] performed a literature review of 45 reports of CAAAF, of which 23 were written in Japanese, and 12 of the 22 reports written in English were from Japan. The authors found that CAAAFs occurred more often in female than in male patients. One third of the patients with CAAAFs were asymptomatic and were usually diagnosed incidentally by cardiac murmur or abnormalities on chest radiographs. The clinical presentation depends on the severity of the left-to-right cardiac shunt. Clinical symptoms, such as fatigue, dyspnea, angina, myocardial ischemia, or myocardial infarction, may appear as the shunt enlarges. The LCX is the origin of the fistula in 18.3% of cases with a coronary artery fistula, with the left anterior descending artery and the right coronary artery accounting for 42% and 50–60% of cases, respectively. The drainage site of the fistula is the CS in 7% of cases [4].

Libertini et al. [5] and Kemmochi et al. [6] reported cases of giant LCX aneurysms with CS fistulas. Almansori and Tamim [7] also reported a case of a giant coronary artery fistula from the LCX to the CS. However, these cases involved a main LCX aneurysm, whereas our case involved a branch aneurysm arising from the LCX. Edwards et al. [8] reported a case of a giant CS with a focal aneurysm secondary to multiple fistulous connections arising from a dilated tortuous LCX. However, the LCX in our patient was of normal caliber and the branch of the LCX was aneurysmal, which were different from these former cases. To our knowledge, this is the first report of the combination of a giant coronary artery branch aneurysm with a fistula to the CS.

Previous reports have also suggested that surgery should be performed for all patients with a CAAAF, even when asymptomatic, due to the risk of future development of cardiac complications, such as rupture, ischemia, or thrombosis [1]. Indeed, Yoshino et al. reported rupture of a coronary artery fistula to the coronary sinus with giant aneurysm of the coronary artery [9]. We followed the strategy recommended in these previous reports. El Nihum et al. recently reported transcatheter embolization of a coronary artery fistula [10]. However, we did not select this procedure as our case involved a coronary branch aneurysm and we considered that cannulation of the fistula would have been difficult and embolization of the LCX main trunk would associated with a risk of ischemia. Moreover, transcatheter embolism would not prevent aneurysm rupture, and coil embolization could affect the blood supply to the normal coronary artery. In our case, there were no coronary side branches arising from the aneurysm that would necessitate coronary bypass grafting, and we therefore performed closure of the orifices in the aneurysm.

The precise mechanisms underlying aneurysm development with a coronary artery fistula are unknown. In our case, pathological examination of both the fistula and aneurysm revealed congenital structural weakness of the coronary artery wall, which may have contributed to the development of the CAAAF. Genetic factors may also be associated with the process of aneurysm formation [1].

We reported the successful surgical treatment of a giant LCX branch aneurysm with a fistula to the CS. Surgical aneurysmectomy should be considered in such cases to avoid fatal aneurysmal complications.

## Data Availability

All data generated or analyzed during this study are included in this published article. Data sharing is not applicable to this article as no datasets were generated or analyzed during the present study.

## Abbreviations

CAAAF: Coronary artery aneurysm with an associated fistula
CS: Coronary sinus
CT: Computed tomography
LCX: Left circumflex artery
LVEF: Left ventricular ejection fraction
